# Management of Low Rectal Cancer Complicating Ulcerative Colitis: Proposal of a Treatment Algorithm

**DOI:** 10.3390/cancers13102350

**Published:** 2021-05-13

**Authors:** Bruno Sensi, Giulia Bagaglini, Vittoria Bellato, Daniele Cerbo, Andrea Martina Guida, Jim Khan, Yves Panis, Luca Savino, Leandro Siragusa, Giuseppe S. Sica

**Affiliations:** 1 Minimally Invasive Surgery, Department of Surgery, Policlinico Tor Vergata, 00133 Rome, Italy; giulia.bagaglini@ptvonline.it (G.B.); Vittoria.bellato@nhs.net (V.B.); daniele.cerbo@alumni.uniroma2.eu (D.C.); andreamartina.guida@ptvonline.it (A.M.G.); leandro.siragusa@alumni.uniroma2.eu (L.S.); giuseppe.sica@uniroma2.it (G.S.S.); 2 Colorectal Surgery Department, Queen Alexandra Hospital, Portsmouth NHS Trust, Portsmouth PO6 3LY, UK; jim.khan@porthosp.nhs.uk; 3 Service de Chirurgie Colorectale, Pôle des Maladies de L’appareil Digestif (PMAD), Université Denis-Diderot (Paris VII), Hôpital Beaujon, Assistance Publique-Hôpitaux de Paris (AP-HP), 100, Boulevard du Général-Leclerc, 92110 Clichy, France; yves.panis@bjn.aphp.fr; 4 Pathology, Department of Biomedicine and Prevention, Policlinico Tor Vergata, 00133 Rome, Italy; luca.savino.88@students.uniroma2.eu

**Keywords:** ulcerative colitis, inflammatory bowel diseases, rectal cancer, surgical management, treatment algorithm

## Abstract

**Simple Summary:**

This article expresses the viewpoint of the authors’ management of low rectal cancer in ulcerative colitis (UC). This subject suffers from a paucity of literature and therefore management decision is very difficult to take. The aim of this paper is to provide a structured approach to a challenging situation. It is subdivided into two parts: a first part where the existing literature is reviewed critically, and a second part in which, on the basis of the literature review and their extensive clinical experience, a management algorithm is proposed by the authors to offer guidance to surgical and oncological practices. This text adds to the literature a useful guide for the treatment of these complex clinical scenarios.

**Abstract:**

Low rectal Carcinoma arising at the background of Ulcerative Colitis poses significant management challenges to the clinicians. The complex decision-making requires discussion at the multidisciplinary team meeting. The published literature is scarce, and there are significant variations in the management of such patients. We reviewed treatment protocols and operative strategies; with the aim of providing a practical framework for the management of low rectal cancer complicating UC. A practical treatment algorithm is proposed.

## 1. Introduction

Chronic inflammation of the large bowel in ulcerative colitis (UC) may set the stage for the development of dysplasia, carcinoma in situ and, finally, invasive adenocarcinoma. Colorectal cancer (CRC) risk is increased by twofoldin UC patients, and this increases with activity and extent of disease, younger age at diagnosis, family history of CRC, associated primary sclerosing cholangitis and, more importantly, disease duration [1]. The estimated risk is 1% after 10 years of disease and 3% and 7–18% after 20 and 30 years, respectively [2,3]. When cancer does develop, its management implies different considerations compared to sporadic cancers, mainly due to the underlying colitis and its field effect on the entire colon. We recently audited our management of non-invasive tumours of the lower rectum in patients with UC. One such patient is hereby presented: a 45-year-old man with a 10 years history of UC presenting with an ultra-low rectal tumour on the anterior rectal wall. Preoperative work-up confirmed a cT1N0M0 adenocarcinoma of the lower rectum, 1 cm from the dentate line. We will use our case as a real-world guide to illustrate the complex decision-making behind the treatment of such patients. The aim of this work is to review the current literature with a particular focus on early-stage, low rectal carcinoma; we also present our treatment algorithm to provide a practical framework for the challenging management of rectal cancers arising in UC.

## 2. Methods

**Figure 1:**
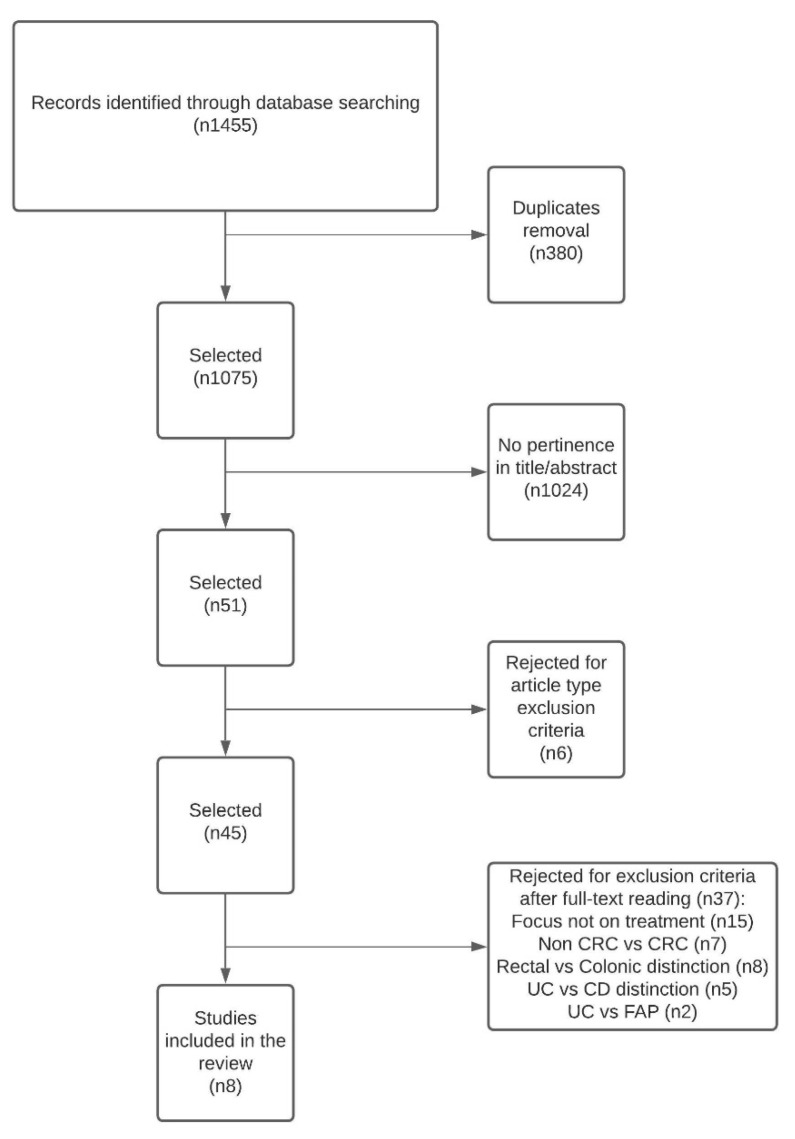
Flow diagram for assessment of studies identified by the search strategy.

A systematic literature search was conducted according to the Preferred Reporting Items for Systematic Reviews and Meta-Analyses (PRISMA) statement (Figure 1). A systematic search of PubMed and Cochrane Library was conducted on 30 August 2020. Keywords used were a combination of the following terms “ulcerative colitis” OR “UC” OR Inflammatory bowel disease” or “IBD” AND “colorectal cancer” OR “colorectal adenocarcinoma” OR “rectal cancer” OR “rectal adenocarcinoma.” Any original paper in English concerning the treatment of rectal cancer in ulcerative colitis was included, inclusive of randomized controlled trials (RCTs), prospective controlled studies, retrospective case series and case reports. Exclusion criteria targeted original articles focusing on UC but not cancer or cancers different from CRC; articles including Crohn’s disease (CD) and not distinguishing between UC and CD; articles including colonic cancers and not distinguishing between the two (rectal and colonic) or not focusing on treatment; narrative review articles, video vignettes, letters, abstracts and conference proceedings. Two independent reviewers originally screened the literature and identified relevant articles. Duplicates were removed, and titles and abstracts were scrutinized for pertinence. Assessment of full papers permitted exclusion of other studies that did not meet inclusion criteria. Any discrepancy among reviewers was solved through discussion and consensus. References of all included articles were checked for additional paper identification. Considered endpoints were treatment modalities, operative strategies, functional and survival outcomes. Particular attention was given to early-stage, low rectal adenocarcinoma. Current national and international guidelines were reviewed critically. Findings are presented, explained and discussed.

The evidence and recommendations cited in this text are reported according to Oxford Centre for Evidence-based Medicine [4].

## 3. Results of Literature Search

Literature search initially identified 1455 studies. Only eight studies fulfilled all criteria and were included in the review (Table 1). Only two studies specifically addressed low rectal cancer. The results of these studies are presented pragmatically in a treatment-related consequential manner.

## 4. Treatment of Rectal Adenocarcinoma Complicating Ulcerative Colitis

The treatment of colorectal adenocarcinoma developing in patients suffering from UC follows much of the same principles applied to sporadic adenocarcinoma with one exception: the indication in these patients is to remove the entire colon and rectum, possibly performing a restorative proctocolectomy [13,14] (EL5). The main reason behind these guidelines is the high risk of metachronous (and hidden synchronous) cancers due to the “field effect” of the disease [15,16,17,18,19] (EL2). Recent reports suggest a more tailored therapy that includes segmental resections or subtotal colectomy (EL4) [20,21]. In particular, they highlight the importance of patient and disease-specific features such as disease duration, extent, clinical and endoscopic activity, biopsy results and patient’s age, performance status and personal priorities. In any case, the decision on the type of surgical resection should be multidisciplinary and taken with the patient.

In the case of rectal cancers in UC, a multidisciplinary team evaluation is mandatory, given the many available treatment options, all of which must be available and eventually integrated to obtain optimal oncologic and functional outcomes. Furthermore, UC patients have been found to have an excess mortality risk from rectal but not colonic cancers, emphasizing the importance of improving treatment for these patients specifically [7,22]. Clinical management includes radiotherapy, chemotherapy, a combination of the two (in both neoadjuvant and adjuvant setting) and various surgical procedures, with different purposes (radicality, organ conservation, functional preservation, etc.). However, even though UC patients should be subject to special considerations due to the unique clinical picture they present, the specific issue of rectal cancer management in UC suffers from a relative paucity of literature (Table 1).

In general, radical surgery with total mesorectal excision is standard of care for early localised disease (with the possible exception of T1N0, which may be treated with local excision), while a neoadjuvant chemo radiotherapeutic treatment is advocated for locally advanced disease (>T2 and/or N+) [23]. Clearly, if the whole colon is removed along with the rectum and anus, as indicated by current guidelines, the only possible option to avoid permanent ileostomy is a restorative ileo-anal pouch (IPAA) [24].

IPAA involves the transposition of a loop of the small bowel (a very radiosensitive organ) deep into the pelvis, complicating the possibility of using postoperative radiotherapy if needed. Remzi et al. report 26 rectal cancer patients who underwent IPAA after proctocolectomy for rectal cancer in UC [6]. The average distance from the anal verge was not reported. Pouch function was optimal in most patients, and oncologic results were acceptable (one death from metastatic cancer and one local recurrence). They advocate that all patients must undergo TME, high vessel ligation and mucosectomy and conclude that “IPAA can be performed with good prognosis if oncological principles are observed.” Merchea et al. described their series of 41 rectal cancer patients with UC [9]. The majority of their cases were stage I or II diseases located in the mid rectum. Most patients were treated with proctocolectomy and end ileostomy. Only 11 patients underwent IPAA reconstruction (six stapled and five hand-sewn). The location of the cancers was not reported in this series either, but no patient who had undergone neoadjuvant radiotherapy was offered IPAA. The complication rate was 24%, with no major morbidity. One patient had pouch failure, and another one who underwent adjuvant radiotherapy developed radiation enteritis and was diverted. Oncologic results were acceptable. The reported rate of 18% pouch failure (3.6 times the rate for benign indications) and oncological results are in line with other CRC series [25]. They stated that IPAA should be considered in selected patients with favourable tumours (not advanced, not treated with radiotherapy). Similarly, Gorfine et al. report 14 patients with rectal cancer who underwent IPAA with mucosectomy, with good oncologic results (83% 5 years overall survival) [7]. Zmora et al. report seven patients treated with double-stapled IPAA but do not report specifically survival outcomes [8]. Two pouches were excised after RT-induced complications.

**Figure 2:**
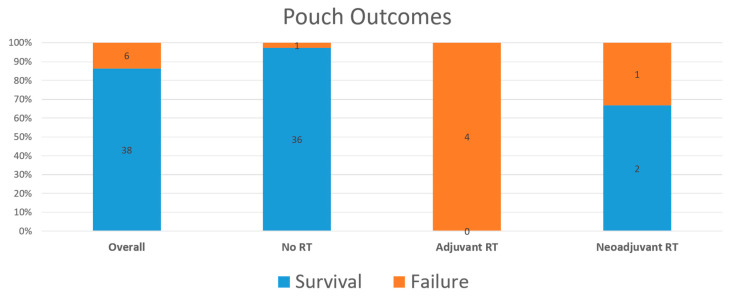
Functional outcomes of IPAA for rectal cancer in ulcerative colitis.

Current guidelines suggest that IPAA should probably be taken into consideration after proctocolectomy for rectal cancer in UC (EL 4). (Table 2; Figure 2).

Nevertheless, as said, not all patients are treated with pan-proctocolectomy (PPC). Khan et al., in a series of 59 patients who underwent either PPC or segmental resection for colorectal cancer in UC, identified three anterior resections and three abdominoperineal resections [21]. Patients in the segmental resection group were significantly older and had shorter standing disease with less severe and less extensive involvement of the colon. None of these patients developed metachronous CRC, and survival outcomes were comparable to patients who underwent PPC.

Radiotherapy (RT) is nowadays standard treatment for sporadic > T2 and/or N+ rectal cancer, especially in the neoadjuvant setting [23,26]. Radiotherapy has the same indication also in UC, although its use implies additional considerations. First, a higher risk of severe acute toxicity in patients with IBD has been reported, albeit with few long-term complications [27,28,29]. Secondarily, evidence for its use in patients who have undergone IPAA is limited to very few cases. Furthermore, a very high rate of pouch failure has been reported in the adjuvant setting due to the predictable effect of radiotherapy on the small bowel used for pouch reconstruction [6,7,8,9,10,25]. Finally, pouch failure is reportedly higher even when radiotherapy is given as neoadjuvant, but overall, the strong rationale and very low-quality evidence indicate that, when considering IPAA, neoadjuvant radiotherapy should be always preferred as the latest European Crohn and colitis organization guidelines indicate [8,10,13,30,31,32] (EL4). (Figure 1).

### 4.1. Low Rectal Cancer

Low rectal cancer is defined as cancer of the rectum arising < 5 cm from the anal margin as measured by rigid sigmoidoscopy [23]. The challenge with the surgical treatment of these tumours is strictly correlated to the possibility of preserving the anal sphincters. For lesions located at or below the mesorectal margin, a 1 cm margin is considered safe enough [26] (EL 3). Tumours that are less than 1 cm from the dentate line generally necessitate abdomino-perineal resection (APR), although selected cases may be eligible for intersphincteric resection and an ultra-low anastomosis. The 1-cm safety rule derives from the historical cohorts showing that distal intramural spread > 1 cm after resection is very uncommon (4–10%) [33,34]. Additionally, in a more recent study, an intramural extension was found to be present in only two out of 108 cases (1.8%) and, in both cases, <0.95 cm [35]. Furthermore, Kuvshinoff et al. demonstrated a similar disease-free survival (DFS) when the distal resection margin was >1 cm or <1 cm from the cancer [36]. In their study, only cancers < 3 mm from the resection margins suffered from worse DFS, and in a similar study, only positive margins were relevant to overall survival [37]. While in sporadic cancers, every effort is made to avoid APR, UC treatment is not standardised, and literature is scarce. Additionally, it is often difficult to point to the exact localization of the lesions. Hotta et al. have analysed 11 patients with very low rectal cancer treated at their institution [12]. They denounce this difficulty, attributed to a high prevalence (89%) of flat mucosal cancer surrounded by chronically inflamed mucosa [38,39,40,41,42,43]. They concluded that the distinction between neoplasia and non-neoplastic tissue is often unclear and increases the potential for distal margin positivity. In their series, nine patients with lesions > 2 cm from the dentate line underwent proctocolectomy, mucosectomy and IPAA, whilst two had APR. No patient received neoadjuvant therapy or postoperative radiotherapy. They reported a cancer-specific 5-year overall survival rate of 100%, but functional results were not reported. They conclude that a restorative operation (with mucosectomy) is feasible in low-lying cancers < 5 cm from the dentate line with good oncologic results.

### 4.2. Ultra-Low Rectal Cancer

Cancers arising from < 2 cm from the dentate line pose significant challenges. For >T1 cancers, intersphincteric resection (ISR), radiotherapy (RT) or their combination seems to be the most reasonable sphincter-saving options. Intersphincteric resection (ISR) consists of resection of the internal anal sphincter via a perineal approach. ISR may be total, with complete internal sphincter resection at the anocutaneous line; subtotal, with resection between the intersphincteric groove and the dentate line and partial, with resection at the dentate line. ISR can also be conducted asymmetrically and limited to one or more quadrants. These techniques offer the opportunity to preserve the anal canal with good oncologic results, even for very low-lying tumours (<1 cm from the dentate line). Salvage comes at the expense of variable and sometimes poor, functional results [44]. Indications mandate that cancer should not extend into the external sphincter and that the preoperative function of the sphincter apparatus is optimal [45].

UC poses additional doubts because when compared with colo-anal anastomosis, the use of an IPAA following ISR likely poses the patient at greater risk for a poor functional outcome. Patients with an ileo-anal pouch are known to have a higher number of evacuations, ranging from 1 to 30 (median 7) per day, and in expert IBD centres, around 5% of pouches are ultimately taken down due to functional derangements and poor quality of life [46,47,48]. This is the main reason why IPAA after ultra-low anterior resection is often not proposed.

In the literature, only two cases exist of cancers < 2 cm from the dentate line that were managed with restorative surgery. One case is in the series by Hotta et al.: a patient with a flat mucosal cancer < 2 cm from the dentate line who underwent IPAA with intersphincteric resection. The cancer was clinically (and pathologically) T1. Distal margins were positive. Nonetheless, regular pouchoscopy did not reveal recurrence. The patient died 81 months later of pancreatic cancer. Another case was described by Inoue et al.: a 59-year-old woman with a 24-year history of UC and advanced (T3N2M0) rectal cancer located 2 cm from the anal verge [10]. The patient underwent neoadjuvant chemoradiotherapy, with a good response and tumour shrinkage (yTNM and distance form verge not reported). Due to her strong desire to avoid permanent stoma, after being fully informed, she underwent IPAA with intersphincteric resection of the posterior quadrant. One year after ileostomy closure (18 months post-IPAA), her pouch function was good, with no disease recurrence.

The above-cited experiences have shown that IPAA is feasible even in the setting of ultra-low rectal cancer when combined with other strategies such as RT or ISR. Nonetheless, evidence is extremely poor, and it should be considered experimental.

Neoadjuvant RT offers the possibility of downstaging, increasing the chance for reconstructive surgery [49,50]. On the other hand, it may be deleterious to sphincter function, especially when combined with a low-lying anastomosis [51]. There is evidence that irradiated sphincters suffer collagen deposition and myenteric plexus damage [52,53]. Even when combined with intersphincteric resection RT seems to be the primary factor involved in poor anal function [54]. RT’s sphincter damaging effect, combined with its effect on pouch survival, probably jeopardize the chances of success and limit its use. Nonetheless, the only case found in the literature was successful [10].

### 4.3. Early Stage Ultra-Low Rectal Cancer

Sporadic T1N0 cancers are amenable to local excision [23,26]. In fact, surgical local excision was reported in a meta-analysis to be equivalent to radical surgery in terms of oncologic end-points (overall survival, disease-free survival, local recurrence-free survival and metastasis-free survival) and superior in terms of perioperative parameters (blood loss, hospital stay, operative time, number of permanent stomas and perioperative deaths) [55,56] (EL1). Several techniques are used for local excision, but Transanal Endoscopic Microsurgery (TEM) has shown superiority over endoscopic submucosal dissection and traditional Trans-Anal Excision (TAE), despite the current absence of randomised trials [57,58,59]. However, the reported differences in outcome between TEM and TAE may be explained by the preferential use of TEM in tumours of the upper and mid rectum and TAE in tumours of the lower rectum (and sometimes the distal mid-rectum), which may be intrinsically more prone to nodal metastases. Nascimbeni et al. found a 3–4 times increase in nodal metastases between low vs. mid and upper rectum, respectively [60,61].

However, only a subset of T1 cancers can be considered adequately treated by local excision (Box 1). In sporadic cancers, if histology confirms a low-risk T1 lesion, no further cancer-specific treatment is needed. On the other hand, in UC, the necessity of prophylactic proctocolectomy remains unchanged.

Box 1 **How histology results guide successive treatment.**

The treatment algorithm for T1 rectal cancers requires the careful evaluation of the histological characteristics of the lesion. In fact, Kikuchi et al. developed a sub-classification of T1s based on the depth of submucosal invasion, which differentiates cancers into “sm1” (slight submucosal invasion from the muscularis mucosa to the depth of 200 to 300 µm), “sm2” (intermediate invasion of 2/3 of the submucosa > 300 µm) and “sm3” (complete invasion of the submucosa near the inner surface of the muscularis propria) [62]. The distinction between sm1, sm2 and sm3 tumours is crucial. In fact, sm1 cancers have a 3% risk of nodal metastases that increases to 8% and 23% for sm2 and sm3 tumours, respectively [61]. In sm1 tumours or in those sm2 cases without worrisome features, radical surgery can be safely omitted [61]. TME is needed for the rest of sm2 and sm3 tumours (EL3). High-risk histological features are: grade 3, tumour budding, vascular, lymphatic or perineural invasion, >3 cm in diameter, occupying > 1/3 of the lumen circumference, < 1 mm microscopic margin [63,64,65]. Patients harbouring these cancers should be counselled in favour of a TME. Data on salvage TME (i.e., performed after local excision) is still partly contradictory, with some reports of no difference compared to primary TME in all aspects (intra-, post-operative and long term) and others of a more difficult procedure with a significantly higher rate of ultimate APR [66,67]. When very low (<1 cm from DL), radical surgery includes APR.

To our knowledge, no specific study exists on the treatment of very low (<2 cm) T1 rectal cancers in UC. Therefore, the optimal treatment strategy is not defined, yet several options can be considered. IPAA with intersphincteric resection is feasible and may yield acceptable outcomes; therefore, it may represent a valid but not validated option. Furthermore, in the only reported case, margins were positive despite ISR. However, a more convenient approach is possible for T1 cancers: local excision, followed by restorative proctocolectomy. This approach has two advantages: it allows for a definitive confirmation of stage and, therefore, in case of the T1sm1, of adequate treatment and avoidance of ISR or even PPC. Histology after local excision may confirm the complete removal of cancer tissue, turn away concerns (and risks) for distal margins in the setting of flat lesions and allow for less aggressive surgery and conservation of the anus without the morbidity of an intersphincteric resection. Local excision may also avoid the need for mucosectomy and offer the possibility of fashioning a double-stapled anastomosis, as the previous scar can be incorporated into the anastomosis without concerns for local recurrence. Last and definitively not the least, in favourable cases, patients can also be spared the morbidity of a TME, which represents a substantial advantage, especially in young men (who represent a large fraction of these cases).

**Figure 3:**
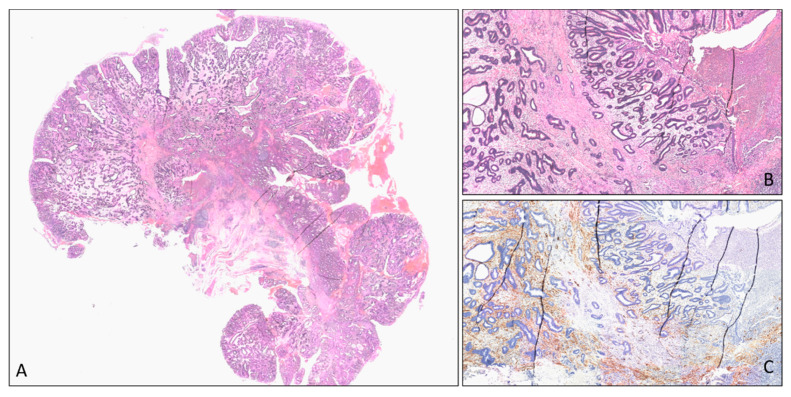
Histologic features of early T1 colorectal carcinoma. (**A**) At low power (Haematoxylin and Eosin, original magnification 10×), the picture shows a malignant sessile polyp. The polypectomy margin is free from neoplasia. At high power (H&E, original magnification 40×), isolated and neoplastic glands invade the upper third of the submucosa (sm1 according to the Kikuchi classification). (**B**) Smooth muscle actin immunohistochemistry stain; (**C**) confirms the presence of glands exceeding the muscolaris mucosae.

**Figure 4:**
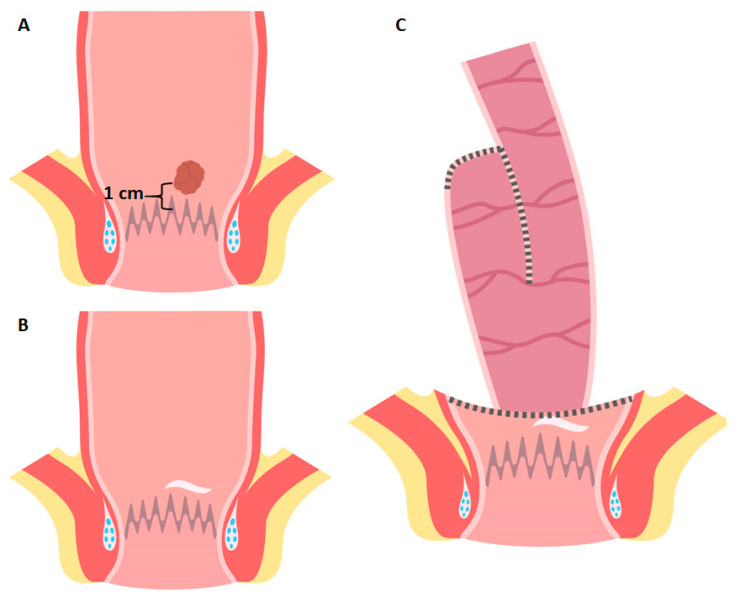
Local excision combined with stapled ileal pouch-anal anastomosis. (**A**) Schematic representation of low rectal cancer, less than 2 cm from the dentate line. (**B**) Residual rectum with local excision scar after first stage surgery. (**C**) Stapled IPAA on the residual rectal cuff at the level of the previous excision.

Our patient with a 10-years history of UC and diagnosis of T1 rectal cancer 1 cm from the dentate line was offered this staged approach involving PPC a full-thickness trans-anal excision of the lesion with a 10 mm margin, requiring the distal resection margin falling on the dentate line anteriorly. Histology confirmed T1, low grade, sm1 cancer with favourable features (Figure 3), and our patient underwent a “bad TME” proctocolectomy (Box 2) and double-stapled IPAA (Box 3) with good functional results (Figure 4).

Box 2 **TME in rectal cancer.**

In the case of T1sm1 low rectal cancer, the risk of nodal metastases is low but not absent (3% in sporadic t1 sm1 cancers), and it would be oncologically safer to excise the mesorectum. Complications of TME include erectile dysfunction in men and decreased lubrication and dyspareunia along with decreased fertility in women; mild symptoms, such as stress and urge incontinence, are also relatively common after TME [68,69]. A “bad TME” is now the preferred approach when performing rectal resection for UC, with the rationale of decreasing sexual dysfunction and urinary sequelae [30] (EL4), even though a retrospective study failed to show a significant decrease in complications with a close rectal dissection compared to a TME dissection plane, but the incidence was more than halved (2.2 vs. 4.5%) [70]. Finally, some series report lower septic complications after mesorectal conservation, which, in turn, may reduce risks of presacral sinus should the anastomosis leak [71,72]. For sm2 lesions (8% risk of nodal metastases), TME can be discussed carefully with the patient, but the advised approach is definitively in favour of radicality. TME is clearly mandatory for any sm3 adenocarcinoma of the rectum.

Box 3 **Stapled or hand-sewn IPAA?**

IPAA can be performed through two main techniques: double-stapled anastomosis (DSA) and mucosectomy, followed by hand-sewn anastomosis (MHSA). Double-stapled anastomosis is accomplished by anastomosing a residual rectal cuff with the ileal pouch with a mechanical circular stapler. In-built in this technique is leaving a residuum of the rectum. Mucosectomy is the excision of all rectal mucosa with the preservation of 1–2 cm of rectal wall to use for subsequent hand-sewn anastomosis. This technique does not leave any rectal mucosa but results in a lower anastomosis. A third technique is gaining popularity: double purse-string single-stapled anastomosis. This type of anastomosis is performed trans-anally with a circular stapler after fashioning a purse-string on both the pouch and the rectal stump [73]. Current guidelines recommend the use of a low-stapled anastomosis due to its marginally superior functional outcomes (EL2). They also advise for the maintenance of a rectal cuff < 2 cm, as a longer cuff poses the patient at higher risk of cuffitis and of new cancer development (EL3). Nevertheless, MHSA shows in most studies results similar to the stapled anastomosis with the exception of nocturnal incontinence, which seems to be increased with mucosectomy [74].

Oncologic results are also similar between the two techniques, and no evidence supports either above the other. In fact, cancers have been found to occur even when mucosectomy is performed [75]. In general, although experts admit to a rationale favouring mucosectomy in the case of low rectal cancer [76] (EL5), quality evidence does not favour either technique (EL2). One possible approach is to keep a low threshold for secondary mucosectomy should dysplasia be evident in follow-up endoscopies and/or biopsies, as is advocated by some groups in the management of Familial Adenomatous Polyposis patients [77].

## 5. Proposal of a Treatment Algorithm

On the premises of what is discussed above, we have developed a treatment algorithm for low tumours of the rectum in UC patients that encompasses diverse scenarios. All options are well established in the management of sporadic rectal cancer. However, given the low level of evidence in UC patients, the proposed scheme should be considered in highly experienced tertiary referral centres, and patients should be informed that pouch failure rates might be higher in this setting.

**Figure 5:**
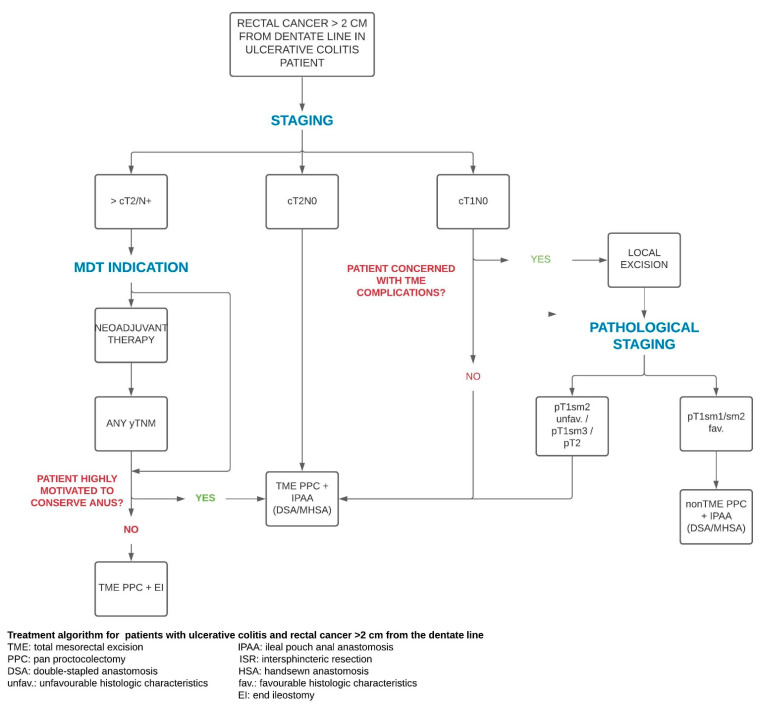
Treatment algorithm for patients with ulcerative colitis and rectal cancer >2 cm from the pectinate line.

UC patients with rectal cancer > 2 cm from the dentate line have a quite linear course that involves upfront IPAA in early disease and neoadjuvant CRT in locally advanced disease (as in sporadic cases) followed by IPAA/PPC with TME. For T1 tumours, the benefits of local excision might be the avoidance of TME (Figure 5).

**Figure 6:**
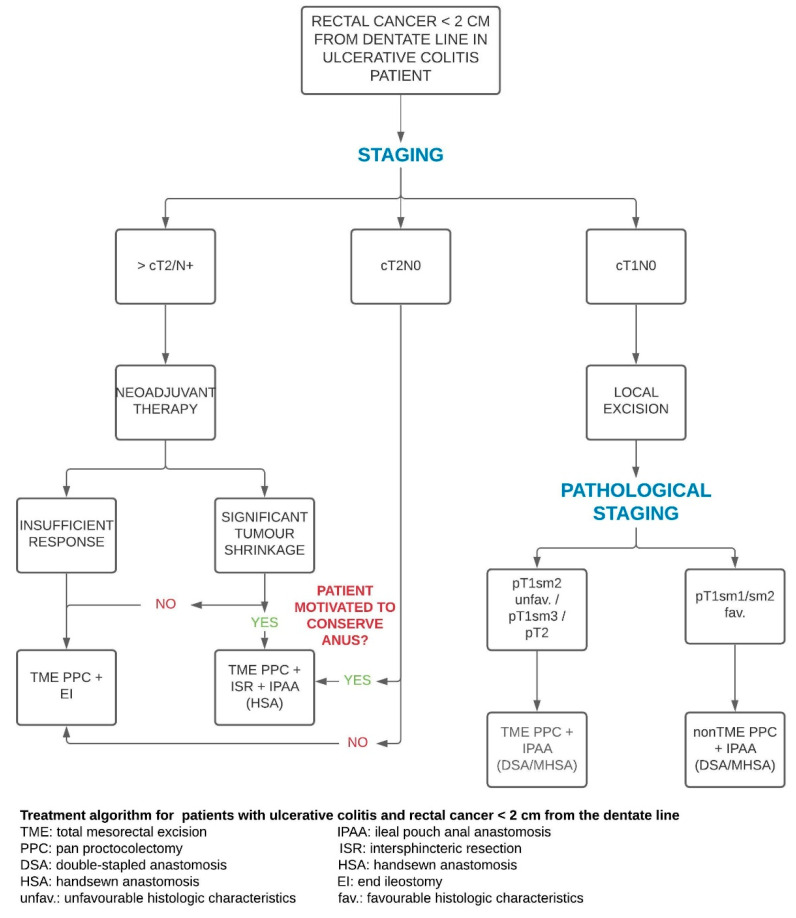
Treatment algorithm for patients with ulcerative colitis and rectal cancer < 2 cm from the pectinate line.

In patients with ultra-low rectal cancer (<2 cm from the dentate line), neoadjuvant CRT is indicated in case of locally advanced cancers, and it might be discussed also for T2 lesions, with the intent of shrinking the tumour and possibly avoiding PPC. Highly motivated and fully informed patients who refuse definitive ileostomy might undergo ISR and IPAA. For cT1 cases, local excision and staged proctocolectomy plus IPAA offer the advantages of maximizing functional outcome in the context of oncological safety. In the case of pT2, patients will be counselled for the need for a TME -PPC/IPAA (Figure 6). The follow-up depends on the index operation.

## 6. Conclusions

Low rectal cancers developing in UC present a clinical challenge. The scarcity of literature makes decision-making particularly difficult. Neo-adjuvant treatment should be considered when appropriate, but it may jeopardize restorative surgery, which should be offered only to highly motivated patients. Sphincter-saving surgery followed by IPAA reconstruction is a viable option in most other instances, especially when the cancer is >2 cm from the dentate line. For very low-lying tumours in highly motivated patients, intersphincteric resection and IPAA could be considered. For T1 sm1 (or even sm2) lesions, a staged approach consisting of local excision, followed by bad TME proctocolectomy and double-stapled IPAA is a strategic option that seems to offer some advantages, including maintaining continence and minimizing morbidity from TME- and mucosectomy-related complications. Pan-proctocolectomy and end ileostomy remain a curative option at the price of loss of faecal continence. A tentative treatment algorithm is hereby proposed.

## Figures and Tables

**Table 1:** Rectal cancer in ulcerative colitis studies.

Publication Year	First Author	Article Type	Patients (*n*)	Stage (n)	Distance from Dentate Line (n)	Radiotherapy (n)	Surgical Procedure (n)	DSA/MHSA (n)
1999	Shimizu [5]	Case Report	1	Not reported	Upper rectum	Not reported	Subtotal colectomy	Not reported
2002	Remzi [6]	Retrospective case series	26	Stage 0, 7; Stage I, 9; Stage II, 3; Stage III, 7;	Not reported	1 adjuvant CRT → pouch loss	IPAA	10 vs. 16
2004	Gorfine [7]	Retrospective case series	14	Stage I, 9; Stage 2, 2; Stage 3, 3;	Not reported	1 neoadjuvant CRT → pouch survival; 1 adjuvant CRT → pouch loss	IPAA	0 vs. 14
2009	Zmora [8]	Retrospective case series	7	Stage I, 3; Stage II 3, Stage III, 1;	Upper rectum, 3; Mid rectum, 3; Low rectum, 1	1 neoadjuvant CRT → pouch loss; 1 adjuvant CRT → pouch loss	IPAA	7 vs. 0
2012	Merchea [9]	Retrospective case series	41	Stage I, 18; Stage II, 10; Stage III, 10; Stage IV, 3	Upper rectum, 8; Mid rectum, 19; Low rectum, 13; Unknown, 1;	4 neoadjuvant (0 in IPAA group); 14 adjuvant (1 in IPAA group) → 1 pouch loss	IPAA, 11; PPC, 27; APR, 2; Subtotal colectomy, 1	6 vs. 5
2013	Inoue [10]	Case Report	1	Stage III	2 cm	Neoadjuvant CRT	IPAA with ISR	HSA
2017	Tsuchiya [11]	Case report	1	Stage I	Upper rectum	Not used	PPC	Not reported
2018	Hotta [12]	Retrospective case series	11	Stage 0, 5; Stage I, 2; Stage III, 2; Unknown, 2	Lower rectum	Not used	IPAA, 8; IPAA with ISR, 1; PPC, 2	0 vs. 9

**Table 2:** Oncologic results of surgery for rectal cancer in ulcerative colitis.

Paper	Patients (*n*)	Follow-up	Oncologic Outcomes
Remzi 2002 [6]	26	Mean 6.1 years; 2 lost to fu	95.8% overall survival
Gorfine 2004 [7]	14	Not specified	83.5% estimated overall survival at 5 years
Merchea 2012 [9]	41	Median 4.4 years	62% overall survival
Inoue 2013 [10]	1	1 year	Disease free survival
Tsuchiya 2017 [11]	1	2 years	Disease free survival
Hotta 2018 [12]	11	5 years	100% disease free survival

## Data Availability

Data supporting the main text and conclusions of this paper is available online in the form of previously published manuscripts.
